# Lithium promotes long-term neurological recovery after spinal cord injury in mice by enhancing neuronal survival, gray and white matter remodeling, and long-distance axonal regeneration

**DOI:** 10.3389/fncel.2022.1012523

**Published:** 2022-11-11

**Authors:** Zeynep Balçıkanlı, Irem Culha, Pelin Dilsiz, Mehmet Serif Aydin, Nilay Ates, Mustafa Caglar Beker, Saltuk Bugra Baltaci, Halil I. Koc, Ahmet Yigitbasi, Mustafa Gündogar, Thorsten R. Doeppner, Dirk M. Hermann, Ertugrul Kilic

**Affiliations:** ^1^Department of Physiology, Regenerative and Restorative Medical Research Center, Istanbul Medipol University, Istanbul, Turkey; ^2^Department of Physiology, School of Medicine, Istanbul Medipol University, Istanbul, Turkey; ^3^Research Institute for Health Sciences and Technologies (SABITA), Istanbul Medipol University, Istanbul, Turkey; ^4^Department of Pharmacology, School of Medicine, Istanbul Medipol University, Istanbul, Turkey; ^5^Department of Hematology, Medical Faculty, Trakya University, Edirne, Turkey; ^6^Department of Endodontics, Faculty of Dentistry, Istanbul Medipol University, Istanbul, Turkey; ^7^Department of Neurology, University Hospital Gießen, Göttingen, Germany; ^8^Department of Neurology, University Hospital Essen, University of Duisburg-Essen, Essen, Germany

**Keywords:** axonal plasticity, axonal regeneration, motor coordination, neurological recovery, spinal cord hemitransection, spinal cord trauma

## Abstract

Spinal cord injury (SCI) induces neurological deficits associated with long-term functional impairments. Since the current treatments remain ineffective, novel therapeutic options are needed. Besides its effect on bipolar mood disorder, lithium was reported to have neuroprotective activity in different neurodegenerative conditions, including SCI. In SCI, the effects of lithium on long-term neurological recovery and neuroplasticity have not been assessed. We herein investigated the effects of intraperitoneally administered lithium chloride (LiCl) on motor coordination recovery, electromyography (EMG) responses, histopathological injury and remodeling, and axonal plasticity in mice exposed to spinal cord transection. At a dose of 0.2, but not 2.0 mmol/kg, LiCl enhanced motor coordination and locomotor activity starting at 28 days post-injury (dpi), as assessed by a set of behavioral tests. Following electrical stimulation proximal to the hemitransection, LiCl at 0.2 mmol/kg decreased the latency and increased the amplitude of EMG responses in the denervated hindlimb at 56 dpi. Functional recovery was associated with reduced gray and white matter atrophy rostral and caudal to the hemitransection, increased neuronal survival and reduced astrogliosis in the dorsal and ventral horns caudal to the hemitransection, and increased regeneration of long-distance axons proximal and distal to the lesion site in mice receiving 0.2 mmol/kg, but not 2 mmol/kg LiCl, as assessed by histochemical and immunohistochemical studies combined with anterograde tract tracing. Our results indicate that LiCl induces long-term neurological recovery and neuroplasticity following SCI.

## Introduction

Spinal cord injury (SCI) defines the traumatic disruption of neuronal networks at any spinal cord level (Zhang S. et al., 2018), inducing sensorimotor deficits and giving rise to long-term functional impairments (Carelli et al., 2019). Following the acute injury, secondary pathomechanisms, including free radical formation, ischemia, and inflammation, further aggravate the primary tissue damage, inducing secondary neuronal degeneration and spinal cord atrophy (Volarevic et al., 2013; Boyce and Mendell, 2014; Zhang D. et al., 2018). Surgical, pharmacological, and rehabilitative interventions have been studied regarding their capacity of improving functional SCI recovery, so far with limited success (Elizei and Kwon, 2017; Nori et al., 2017). There is an urgent need of treatments that enhance spinal cord remodeling and clinical outcome.

Lithium chloride (LiCl) (in the following referred to as lithium) is widely used for the treatment of bipolar mood disorder. Lithium revealed neuroprotective and anti-inflammatory effects in several neurodegenerative conditions (Young, 2009; Chiu and Chuang, 2011; Huo et al., 2012; Li et al., 2015; Lauterbach, 2016). Thus, lithium was shown to improve neurological recovery and neuronal injury in an ischemic stroke model (Nonaka and Chuang, 1998), to prevent hippocampal neurodegeneration and cognitive decline in an Alzheimer’s disease model (De Ferrari et al., 2003) and to decrease neurodegeneration in a Parkinson’s disease model (Youdim and Arraf, 2004). Recent studies revealed that lithium elevates central nervous system (CNS) levels of brain-derived neurotrophic factor (BDNF) and its receptor TrkB (Dwivedi and Zhang, 2014; Abdanipour et al., 2019), which promote neuroplasticity and neurological recovery in the injured spinal cord (Keefe et al., 2017). Despite these findings, previous studies focused on the neuroprotective effects of lithium in the early SCI phase. These studies, all done in rats, revealed that lithium reduced structural spinal cord damage and neurological deficits *via* mechanisms involving autophagy activation and antiinflammation (Kim et al., 2017; Liu et al., 2017; Zhang D. et al., 2018; Abdanipour et al., 2019).

The effect of lithium on long-term neurological recovery and neuroplasticity remained to be clarified. This study aimed to evaluate the dose-dependent effect of lithium on disrupted motor coordination after long-term SCI and investigate the effect of long-term lithium administration on post-injury tissue damage, cell survival, and axonal regeneration. We herein exposed mice to spinal cord hemitransection and evaluated lithium’s actions on (a) motor coordination recovery using a detailed test battery that involved the Basso mouse scale (BMS), horizontal ladder rung, and open field tests, (b) functional electromyography (EMG) responses of the denervated hindlimb following electrical spinal cord stimulation rostral to the lesion, (c) neuronal degeneration and gray and white matter remodeling using histochemical and immunohistochemical techniques, and (d) the regeneration of anterogradely labeled long-distance spinal cord axons. Our data reveal a neurological recovery- and neuronal plasticity-promoting action of low-dose (0.2 mmol/kg), but not high-dose (2 mmol/kg) lithium which persisted over up to 56 days post-SCI (dpi). The data shown here will provide an understanding of the effects of long-term lithium treatment on motor coordination, neuronal survival, and axonal plasticity in the post-injury spinal cord and offer an alternative and novel approach to the existing treatment options for SCI.

## Materials and methods

### Animals and experimental groups

The experiments were performed in accordance with National Institutes of Health (NIH) guidelines for the care and use of laboratory animals and were approved by the local government authorities (Istanbul Medipol University Animal Research Ethical Committee). All animals were housed at 22–24°C in a constant 12-h light (07:00–19:00 h) and dark (19:00–07:00 h) cycle. For behavioral analysis and immunohistochemistry, a total of 48 male Balb/c mice (9–12 week-old) were randomly divided into three groups that were intraperitoneally treated with (i) vehicle (0.9% isotonic saline), (ii) 0.2 mmol/kg lithium (dissolved in 0.9% isotonic saline) or (iii) 2 mmol/kg lithium (dissolved in 0.9% isotonic saline) (*n* = 16 mice/group). To detect plasma lithium concentrations, the same treatment protocol was applied to total of 54 male Balb/c mice (9–12 week-old) (*n* = 3 mice/group/time point).

### Analysis of plasma lithium concentrations

Vehicle, 0.2 mmol/kg or 2 mmol/kg lithium was administered to the animals. A total of 30 min, 3, 6, 12, or 24 h after treatment, the mice were sacrificed, and the blood samples were obtained and centrifuged at 10,000 rpm for 10 min at room temperature (22°C). Sera were collected and stored at −80°C to measure serum lithium concentrations in a clinical biochemistry laboratory (Centro, Istanbul, Turkey) using Roche Cobas C501 instrument (Basel, Switzerland), calibrated (Roche, Kit No: 10759350 190), and analyzed (Kit No: 04679598 190) (Ates et al., 2022).

### Induction of spinal cord injury

Animals were anesthetized using 1% isoflurane (30% O_2_, remainder N_2_O). Rectal temperature was maintained between 36.5 and 37.0°C using a feedback-controlled heating system. Mice were placed in a stereotaxic instrument (World Precision Instruments, FL, USA). SCI was performed as previously described with minor modifications (Chen et al., 2019). Briefly, the skin, subcutaneous fascia layer, muscle, and muscle tendons from T9 to T11 and the bilateral vertebral plate of T10 were removed to expose the T10 segment of the spinal cord. Then, a hemisection was performed on the right side of the spinal cord using a 26G syringe tip. After induction of injury, superficial and deep back muscles and the skin were sutured. All mice exhibited complete paralysis of their right hindlimbs immediately after the surgery.

### Lithium administration

Lithium doses were selected based on a previous study (Doeppner et al., 2017; Ates et al., 2022). Animals were intraperitoneally (i.p.) treated with normal saline (vehicle) or LiCI (0.2 mmol/kg or 2 mmol/kg dissolved in normal saline; L4408; Sigma-Aldrich, St. Louis, MO, USA) at 24-h intervals starting 24 h after SCI for 56 days. Animals were sacrificed at 56 dpi (Supplementary Figure 1).

### Behavioral tests

Functional neurological recovery was assessed using the following tests:

#### Basso mouse scale

The BMS is an accurate method used to evaluate motor functions after SCI (Basso et al., 2006). The scale consists of a 0–9 point rating scale, which evaluates the hindlimb motor functions. The point scale was divided as follows: 0 = no ankle movement, 1 = slight ankle movement, 2 = extensive ankle movement, 3 = plantar placing of the paw or dorsal stepping, 4 = occasional plantar stepping, 5 = frequent or consistent plantar stepping, no coordination; 6 = frequent or consistent plantar stepping with some coordination; 7 = frequent or consistent plantar stepping with severe trunk instability; 8 = frequent or consistent plantar stepping with mild trunk instability; and 9 = frequent or consistent plantar stepping with normal trunk stability (Basso et al., 2006). Animals were observed in an open field chamber for 4 min and blindly evaluated by two different observers, of which mean scores were obtained. BMS scores were determined at 1, 3, 7, 14, 28, and 42 dpi.

#### Horizontal ladder rung walking test

The ladder rung test was adapted from a previous study (Farr et al., 2006). The apparatus consists of two 69.5 cm × 15 cm plexiglass walls each containing 121 holes with 0.20 cm diameter placed 3 cm apart to allow mice to walk through the ladder without hesitation. The ladder has 0.10 cm diameter metal bars that are 0.50 cm apart. Before testing, the animals were acclimated to the apparatus by allowing them to freely walk through the ladder for 10 min. After acclimation, the mice were trained to walk through the metal ladder. Animals were recorded using a high-speed camera and correct foot placement was assessed from the recordings. Correct foot placement was defined as the contact of the hindlimb to the rung with either palm or digits around the rung or a weight-bearing step on the rung. Percent of correct foot placement was measured (correct foot placement × 100/total steps). Horizontal ladder rung tests were obtained at baseline and at 3, 14, 28, and 42 dpi.

#### Open field test

The open field test evaluates spontaneous locomotor activity and exploration behavior of animals in a circular arena (diameter = 150 cm) with a white plastic floor, surrounded by a 35 cm high sidewall made of white polypropylene (Kilic et al., 2014; Kelestemur et al., 2020). The arena was divided into three sections, including an outer wall zone (17.7% of the diameter, close to the wall), an intermediate transition zone (32.3% of the diameter), and an inner zone (50% of the diameter, the center of the arena). Each animal was released near the wall and observed for 10 min using an electronic imaging system (ANY-maze^®^; Stoelting Co., Wood Dale, IL, USA). The resting and progressing time, the time spent in each of the three zones, and the rotations were recorded. Open field tests were performed at baseline and at 3, 14, 28, and 42 dpi.

### Electromyography

Electrophysiological recovery was assessed by the latency and amplitude of gastrocnemius muscle contraction after stimulating the spinal cord above the site of the injury area at the T7 level. At 56 dpi, mice were anesthetized and the hair over the right hindlimb gastrocnemius muscle was shaved. A small skin incision was made to expose the ipsilesional gastrocnemius muscle, in which positive and negative platinum electrodes were inserted. In order to stimulate the spinal cord, laminectomy was performed at the T7 level and the stimulating electrode was inserted into the ipsilesional hemicord, while the ground electrode was placed just below the stimulating electrode above the injury site. Stimulations were performed using 2 V pulses with 10 repeats at 0.2 Hz. Data were obtained using the PowerLab data acquisition system with LabChart 8 software (ADInstruments, Dunedin, New Zealand). The latency, duration, and amplitude of evoked EMG potentials in the gastrocnemius muscle were measured.

### Evaluation of spinal cord remodeling and atrophy

For histochemical analysis, mice were deeply anesthetized and perfused with 4% paraformaldehyde (PFA) at 56 dpi. A 1 cm spinal cord sample centered around the hemitransection, was harvested and dehydrated in 30% sucrose solution at 4°C. In caudal to rostral direction, 50 μm-thick coronal sections were serially collected on glass microscope slides using a cryostat (CM1950, Leica Biosystems, Wetzlar, Germany). To evaluate spinal cord atrophy, Cresyl violet staining was performed on spinal cord sections from the level of T8 (Rostral level) and T13 (Caudal level) according to a standard protocol (Beker et al., 2015; Kelestemur et al., 2020). Sections were imaged using a stereo zoom microscope (AxioZoom.V16, Carl Zeiss, Jena, Germany) and besides gray matter and the dorsal, lateral, and ventral funiculus of the white matter was outlined blindly using Zen Blue software (Carl Zeiss, Germany). Ipsilateral areas were calculated as percent of contralateral areas (Caglayan et al., 2022).

Next, we examined the density of surviving neurons and reactive astrocytes. For this purpose, sections from the spinal cord segments were postfixed in 4% paraformaldehyde (PFA)/0.1 M phosphate-buffered saline (PBS), washed and immersed for 1 h in 0.1 M PBS containing 0.3% Triton X-100 (PBS-T)/10% normal goat serum (G9023; Sigma-Aldrich). Sections were incubated overnight at 4°C with Alexa Fluor 647 conjugated monoclonal rabbit anti-neuronal nuclear protein (NeuN; 1:100; 62,994; Cell Signaling, Beverly, MA, USA) and Alexa Fluor 555 conjugated monoclonal mouse anti-glial fibrillary acidic protein (GFAP) (1:100; 3,656; Cell Signaling) antibody. Sections were analyzed using a laser scanning confocal microscope (LSM780, Carl Zeiss) by evaluating regions of interest (ROI) measuring 62,500 μm^2^ in the dorsal, intermediate and ventral horns. Cells were counted blindly by two different observers in the ipsilesional and contralesional spinal cord. Mean values were calculated for both counting (Caglayan et al., 2019). Data were presented as the ratio of numbers determined in the ipsilesional and contralesional hemicords.

### Tract tracing of axonal regeneration

To detect the axonal plasticity induced by lithium treatment, we traced axons of the lateral spinothalamic tract. Animals were anesthetized at 42 dpi and laminectomy was performed at the L2 level to expose the dorsal segment of the spinal cord (Caglayan et al., 2019). Under visual inspection, 1 μl biotinylated dextran amine (BDA, 10,000 MW; D1956; Invitrogen, Waltham, MA, USA) diluted in 0.01 M PBS was bilaterally injected 0.3 mm lateral to the midline and 0.7 mm deep to the dorsal surface using a Hamilton syringe connected to a glass tip. The syringe tip remained inside the injection area for 1 min to prevent backflow. After 14 days, animals were sacrificed by transcardial perfusion with 4% PFA and coronal sections were collected. Then, sections were washed with PBS, blocked with 10% normal goat serum in PBS for 1 h at room temperature, incubated with Alexa Fluor 555 conjugated streptavidin (S21391; Invitrogen) for 90 min at room temperature and analyzed using confocal microscopy (LSM780, Carl Zeiss). The number of axons crossing the rostral and caudal spinal cord segments were blindly counted in the lateral spinothalamic tract of the ipsilesional and contralesional hemicord in ROI measuring 62,500 μm^2^.

### Statistical analysis

Statistical analysis was performed using SPSS (version 15; SPSS Inc., Chicago, USA) software. Data were evaluated by one-way analysis of variance (ANOVA) followed by least-significant difference (LSD) tests and expressed as mean ± standard deviation (SD). Throughout the study, *p*-values < 0.05 were considered statistically significant.

## Results

### Plasma lithium concentrations following low-dose and high-dose lithium administration

Lithium has a therapeutic range between 0.6 and 1.2 mmol/L in humans (Singer and Rotenberg, 1973; Jope, 1999). Based on our results, we investigated the time and dose-dependent serum concentration of lithium. Although 2 mmol/kg LiCl was previously shown to have neuroprotective activity after ischemic stroke (Ates et al., 2022), we observed that 0.2 mmol/kg LiCl had a restorative effect after SCI. Our findings also imply that serum lithium concentration decreases below <0.05 when measured 6 h after their administration in 0.2 mmol/kg and 12 h in the 2 mmol/kg group (Table 1).

**TABLE 1:** Serum lithium concentrations measured by ICP-MS and shown in mmol/L.

Vehicle	30 min	1 h	3 h	6 h	12 h	24 h
	
	N/A	N/A	N/A	N/A	N/A	N/A
**0.2 mmol/kg**	0.18 ± 0	0.12 ± 0.04	0.09 ± 0.03	<0.05	<0.05	<0.05
**2 mmol/kg**	0.88 ± 0.12	0.45 ± 0.05	0.39 ± 0.09	0.15 ± 0.04	<0.05	<0.05

N/A, not applicable. Data are expressed as mean ± SD (*n* = 3 mice/for each time point).

### Low-dose lithium induces long-term motor coordination recovery

**FIGURE 1:**
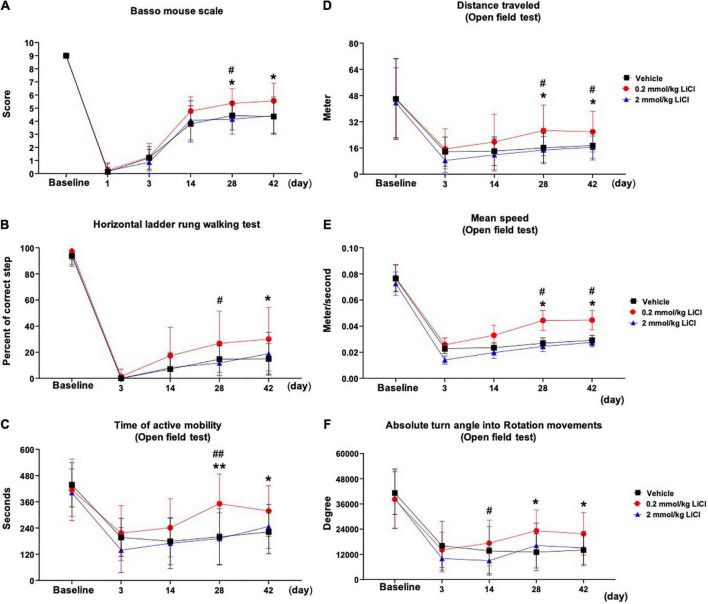
Low-dose lithium enhances motor coordination recovery and spontaneous locomotor activity following spinal cord injury (SCI). Motor performance evaluated by **(A)** the Basso mouse scale and **(B)** the horizontal ladder rung walking test of mice exposed to right-sided spinal cord hemitransection at the T10 level, which were treated with vehicle or lithium (0.2 or 2 mmol/kg/day) for 56 days. Spontaneous motor activity evaluated by **(C)** the time of active mobility, **(D)** the total distance traveled, **(E)** mean speed, and **(F)** rotation movements in the open field test of the same mice exposed to spinal cord hemitransection, which were treated with vehicle or lithium (0.2 or 2 mmol/kg/day) for 56 days. Data are expressed as mean ± SD values (*n* = 16 animals/group). *p < 0.05/^**^p < 0.01 for 0.2 mmol/kg LiCl vs. vehicle; ^#^*p* < 0.05/^##^*p* < 0.01 for 0.2 mmol/kg LiCl vs. 2 mmol/kg LiCl.

To evaluate the effects of lithium on motor coordination, we evaluated ankle movement, plantar placement of the paw with or without a weight support, plantar stepping, gait, and trunk stability in mice exposed to SCI using the BMS. Post-SCI, spontaneous motor movements were absent in ipsilesional (i.e., right-sided) hindlimbs at 1 dpi in all groups (Figure 1A). From 3 to 42 dpi, spontaneous motor movements returned, but directed movements of paretic hindlimbs, stepping and gait were severely impaired in vehicle treated mice (Figure 1A). Lithium at 0.2 mmol/kg, but not 2.0 mmol/kg significantly enhanced motor coordination performance in the BMS at 28 and 42 dpi compared with vehicle (Figure 1A). Likewise, 0.2 mmol/kg lithium significantly increased motor coordination performance in the horizontal ladder rung test, which is widely used post-SCI (Emerick and Kartje, 2004; Ajao et al., 2012; Adkins et al., 2015), at 28 or 42 dpi compared with 2.0 mmol/kg lithium or vehicle, respectively (Figure 1B). As a consequence of the hindlimb impairment, spontaneous locomotor activity assessed by the time of active mobility, the total distance covered, mean speed, and rotation movements in the open field test were markedly reduced post-SCI (Figures 1C–F). Lithium at 0.2 mmol/kg significantly increased the time of active mobility, the total distance covered, mean speed, and rotation movements at 28 and 42 dpi compared with vehicle (Figures 1C–F). Hence, sustained motor coordination recovery was noted in mice receiving 0.2 but not 2.0 mmol/kg lithium.

### Low-dose lithium enhances functional electromyography responses evoked by spinal cord stimulation

**FIGURE 2:**
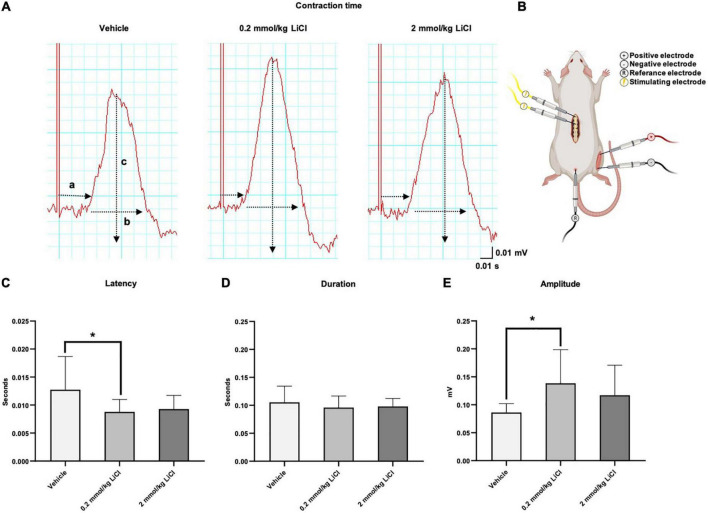
Low-dose lithium enhances functional EMG responses evoked by spinal cord stimulation. **(A)** Representative electromyograms (EMG) obtained in the right-sided gastrocnemius muscle after electrical stimulation of the spinal cord of mice exposed to spinal cord hemitransection, which were treated with vehicle or lithium (0.2 or 2 mmol/kg/day) for 56 days (a: Latency, b: Duration, c: Amplitude). **(B)** Schematic illustration of the electrical stimulation site at the T7 level of the spinal cord and the EMG recording site in the gastrocnemius muscle. After lithium treatment at 0.2 mmol/kg/day but not 2 mmol/kg/day, **(C)** the latency of the evoked potential decreased, **(D)** the duration of the potential remained unchanged and **(E)** the amplitude increased. Data are mean ± SD values (*n* = 16 animals/group). *p < 0.05 for 0.2 mmol/kg LiCl vs. vehicle.

To further characterize the effects of lithium on functional motor recovery, we next recorded EMG responses of the gastrocnemius muscle (Figure 2A) following electrical stimulation of the spinal cord rostral to the lesion site at 56 dpi (Figure 2B). Lithium at 0.2 mmol/kg significantly reduced the latency of the evoked EMG potential (Figure 2C) without influencing its duration (Figure 2D), and significantly increased the EMG amplitude (Figure 2E). Lithium at 2.0 mmol/kg did not influence evoked EMG responses (Figures 2C–E). Collectively, lithium improved functional EMG responses at the 0.2 mmol/kg dose.

### Low-dose lithium prevents spinal cord atrophy rostral and caudal to the lesion

**FIGURE 3:**
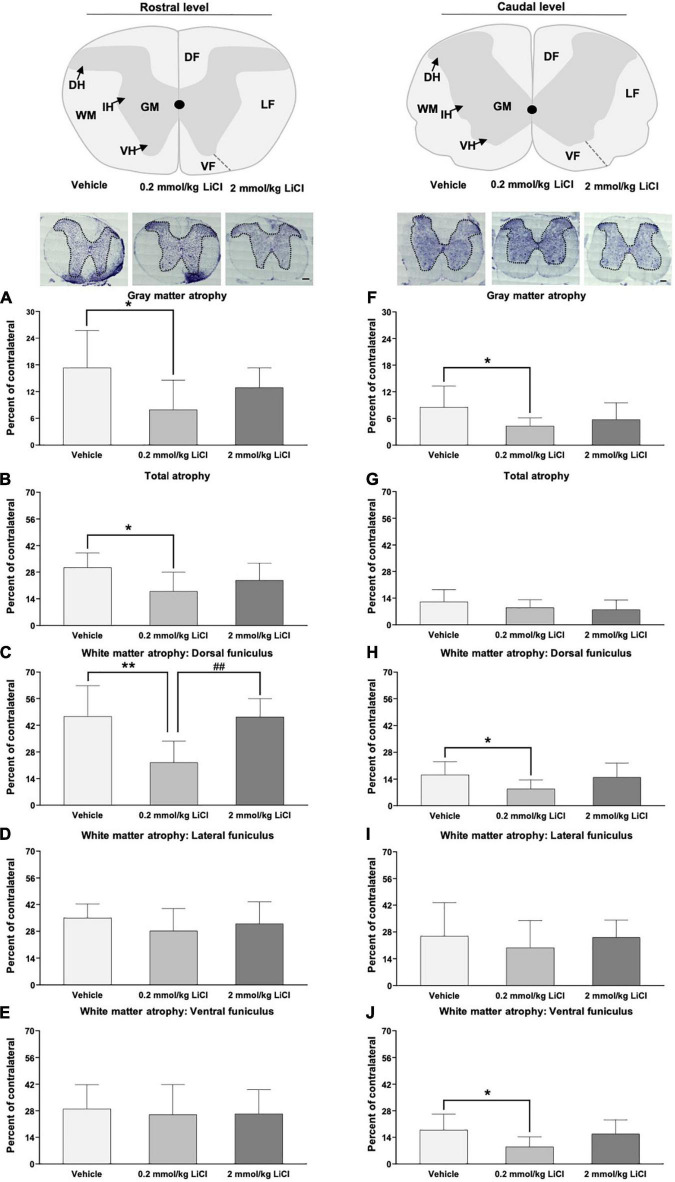
Low-dose lithium reduces spinal cord atrophy rostral and caudal to the hemitransection. **(A,F)** Gray matter atrophy, **(B,G)** total atrophy, **(C,H)** white matter atrophy of the dorsal funiculus, **(D,I)** white matter atrophy of the lateral funiculus, and **(E)** white matter atrophy of the ventral funiculus assessed by Cresyl violet staining at **(A–E)** the rostrocaudal level above the hemitransection (“Rostral level”) and **(F–J)** the rostrocaudal level below the hemitransection (“Caudal level”) of SCI mice, which were treated with vehicle or lithium (0.2 or 2 mmol/kg/day) for 56 days. Representative spinal cord sections are also shown. WM, white matter; GM, gray matter; DH, dorsal horn; IH, intermediate horn; VH, ventral horn; DF, dorsal funiculus; LF, lateral funiculus; and VF, ventral funiculus. Data are mean ± SD values (*n* = 16 animals/group). *p < 0.05/**p < 0.01 for 0.2 mmol/kg LiCl vs. vehicle; ^##^*p* < 0.01 for 0.2 mmol/kg LiCl vs. 2 mmol/kg LiCl. Scale bars, 200 μm.

To examine structural correlates of motor coordination recovery, cresyl violet staining was used to analyze gray and white matter atrophy rostral and caudal to the spinal cord lesion. In mice receiving vehicle, significant gray and white matter atrophy was noted in the injured hemicord both rostral (Figures 3A–E) and caudal (Figures 3F–J) to the lesion site at 56 dpi. Rostral to the spinal cord lesion, the white matter atrophy was most pronounced in the dorsal funiculus (Figures 3C–E), while caudal to the spinal cord lesion, spinal cord atrophy more similarly affected the dorsal, lateral and ventral funiculi (Figures 3H–J). Lithium at 0.2 mmol/kg, but not 2.0 mmol/kg significantly reduced gray matter atrophy rostral and caudal to the spinal cord lesion (Figures 3A,F), reduced white matter atrophy in the dorsal funiculus rostral and caudal to the lesion (Figures 3C,H) and reduced white matter atrophy in the ventral funiculus caudal to the lesion (Figure 3J). Hence, lithium at 0.2 mmol/kg protected against spinal cord degeneration.

### Low-dose lithium prevents neuronal degeneration in the dorsal and ventral horns caudal to the lesion

**FIGURE 4:**
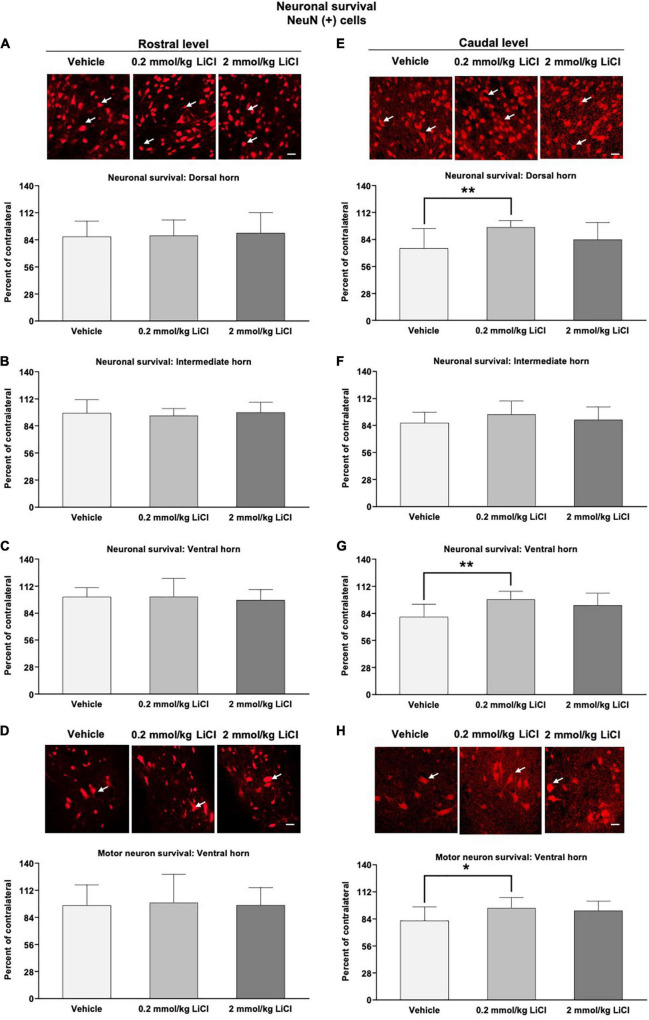
Low-dose lithium prevents neuronal degeneration in the dorsal and ventral horns caudal to the hemitransection. Neuronal survival in **(A,E)** the dorsal horn, **(B,F)** the intermediate horn, and **(C,G)** the ventral horn of the spinal cord, as well as **(D,H)** survival of large-sized motor neurons in the spinal cord ventral horn assessed by NeuN immunohistochemistry at **(A–D)** the rostrocaudal level above the hemitransection (“Rostral level”) and **(E–H)** the rostrocaudal level below the hemitransection (“Caudal level”) of SCI mice, which were treated with vehicle or lithium (0.2 or 2 mmol/kg/day) for 56 days. Representative NeuN stainings are also shown. Data are mean ± SD values (*n* = 16 animals/group). *p < 0.05/**p < 0.01 for 0.2 mmol/kg LiCl vs. vehicle. Please note that white arrows indicate representative neurons. Scale bars, 50 μm.

We subsequently evaluated the survival of spinal cord neurons rostral and caudal to the hemitransection by immunohistochemistry for the neuronal marker NeuN. SCI markedly reduced the density of NeuN^+^ neurons in the ipsilesional dorsal and ventral horns caudal to the hemitransection at 56 dpi (Figures 4A–H). Lithium at 0.2 mmol/kg but not at 2 mmol/kg significantly increased the density of surviving NeuN^+^ neurons in the ipsilesional dorsal and ventral horns caudal to the hemitransection (Figures 4E,G). This neuroprotection also involved the density of large-sized NeuN^+^ motor neurons, which were increased by lithium (Figure 4H). Rostral to the hemitransection, SCI did not influence the density of NeuN^+^ neurons, and neuronal survival was not influenced by lithium (Figures 4A–D).

### Low-dose and high-dose lithium reduces reactive astrogliosis in the gray matter caudal to the lesion

**FIGURE 5:**
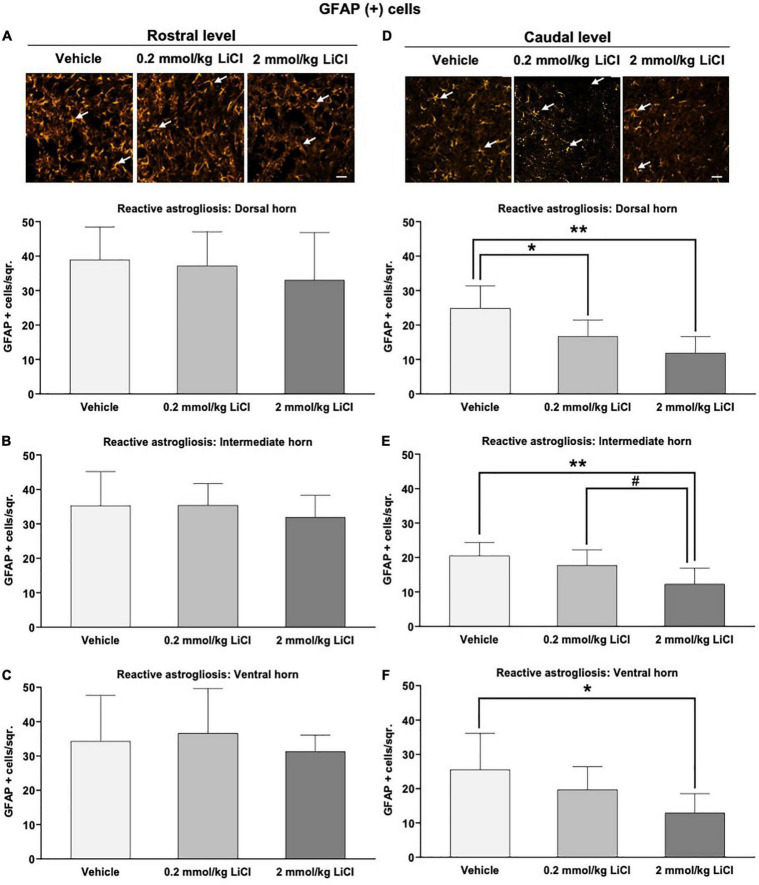
Low-dose and high-dose lithium reduces reactive astrogliosis in the gray matter caudal to the hemitransection. Reactive astrogliosis in **(A,D)** the dorsal horn, **(B,E)** the intermediate horn, and **(C,F)** the ventral horn of the spinal cord assessed by GFAP immunohistochemistry at **(A–C)** the rostrocaudal level above the hemitransection (“Rostral level”) and **(D–F)** the rostrocaudal level below the hemitransection (“Caudal level”) of SCI mice, which were treated with vehicle or lithium (0.2 or 2 mmol/kg/day) for 56 days. Representative GFAP stainings are also shown. Data are mean ± SD values (*n* = 16 animals/group). *p < 0.05/**p < 0.01 for 0.2 mmol/kg or 2 mmol/kg LiCl vs. vehicle; *^#^p* < 0.05 for 2 mmol/kg LiCl vs. 0.2 mmol/kg LiCl. Please note that white arrows indicate representative glia. Scale bars, 50 μm.

We next assessed reactive astrogliosis in the gray and white matter by GFAP immunohistochemistry. Significant reactive astrogliosis was noted in the ipsilesional gray and white matter rostral and caudal to the hemitransection at 56 dpi (Figures 5A–F and Supplementary Figures 2A–F). Lithium at 0.2 and 2 mmol/kg significantly reduced the density of GFAP^+^ astrocytes in the ipsilesional gray matter caudal to the hemitransection (Figures 5D–F), but not the ipsilesional gray matter rostral to the hemitransection (Figures 5A–C). White matter astrogliosis was not influenced by lithium, neither rostral nor caudal to the hemitransection (Supplementary Figures 2A–F).

### Low-dose and high-dose lithium enhances axonal regeneration proximal and distal to the lesion

**FIGURE 6:**
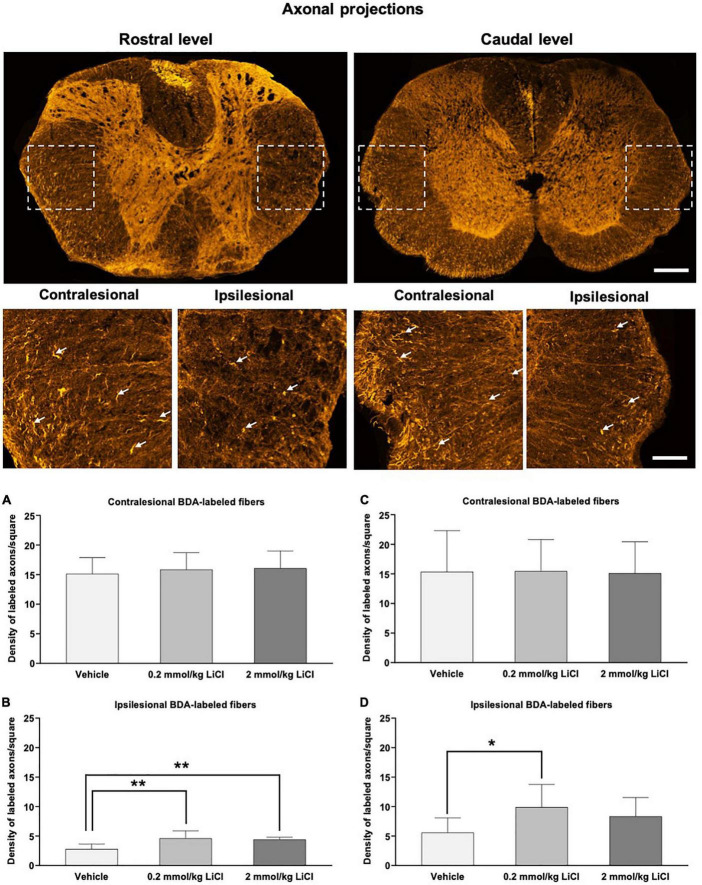
Low-dose and high-dose lithium enhances axonal regeneration proximal and distal to the hemitransection. Representative confocal microscopy images with enlargements of BDA-stained fibers ascending through the injured spinal cord at the rostrocaudal levels above the hemitransection (“Rostral level”) and below the hemitransection (“Caudal level”) of an SCI mouse treated with vehicle, in which BDA had bilaterally been injected into the spinal cord at the L2 level. Quantitative analysis of the density of BDA-labeled fibers in **(A,C)** the contralesional lateral spinothalamic tract and **(B,D)** the ipsilesional lateral spinothalamic tract at **(A,B)** at the rostrocaudal level rostral to the hemitransection (“Rostral level”) and **(C,D)** the rostrocaudal level caudal to the hemitransection (“Caudal level”) of SCI mice, which were treated with vehicle or lithium (0.2 or 2 mmol/kg/day) for 56 days. Data are mean ± SD values (*n* = 16 animals/group). *p < 0.05/**p < 0.01 for 0.2 mmol/kg or 2 mmol/kg LiCl vs. vehicle. Please note that white arrows indicate representative BDA-labeled fibers. Scale bars, 200 μm (lower magnification images)/50 μm (higher magnification images).

To evaluate the effects of lithium on the regeneration of spinal cord axons, we performed tract tracing experiments, in which the anterograde axonal tracer BDA was bilaterally injected into the spinal cord at the L2 level at 42 dpi (Reitmeir et al., 2011, 2012), followed by the evaluation of BDA-labeled axons in both lateral spinothalamic tracts 14 days later, that is, at 56 dpi (Figures 6A–D). The density of BDA-labeled axons in the ipsilesional lateral spinothalamic tract (Figures 6C,D) was markedly lower than that in the contralesional lateral spinothalamic tract (Figures 6A,B), indicating axonal injury in the lesioned hemicord. Lithium at 0.2 mmol/kg significantly increased the density of ipsilesional lateral spinothalamic tract axons caudal to the hemitransection (i.e., proximal to the site of damage) (Figure 6D). Besides, lithium at 0.2 and 2 mmol/kg significantly increased the density of ipsilesional lateral spinothalamic tract axons rostral to the hemitransection (i.e., distal to the site of damage) (Figure 6B). Hence, lithium restored axonal connections, which were disrupted by SCI.

## Discussion

In a model of spinal cord hemitransection, we herein show that lithium, administered i.p. at a dose of 0.2 mmol/kg, enhanced motor coordination recovery over up to 42 dpi, as assessed by the BMS, horizontal ladder rung, and open field tests. Motor coordination improvements were associated with improved functional EMG responses, i.e., an increase in EMG amplitude and a decrease in EMG latency, in the denervated gastrocnemius muscle after spinal cord stimulation rostral to the lesion site. Functional recovery was accompanied by reduced gray and white matter atrophy rostral and caudal to the hemitransection, increased neuronal survival and reduced astrogliosis in the dorsal and ventral horns caudal to the hemitransection, and increased regeneration of long-distance axons proximal and distal to the lesion site, as assessed by histochemical studies combined with anterograde tract tracing at 56 dpi. Lithium at a dose of 2 mmol/kg i.p. did not influence motor coordination recovery, evoked EMG responses, neuronal survival, or spinal cord remodeling. In line with previous studies (Finley, 2016) we conclude that higher dose remained ineffective since lithium has a relatively narrow therapeutic window in which long-term neurological recovery and neuroplasticity are induced.

Previous studies, all performed in rat models, already examined the neuroprotective effects of lithium in the early SCI phase, demonstrating that lithium reduced structural spinal cord damage and motor coordination deficits *via* mechanisms including autophagy activation, antiinflammation and neurotrophic factor (specifically, BDNF) formation (Kim et al., 2017; Liu et al., 2017; Zhang D. et al., 2018; Abdanipour et al., 2019). So far, two studies evaluated lithium’s effects on motor coordination deficits for up to 14 dpi (Kim et al., 2017; Abdanipour et al., 2019) and one study for up to 28 dpi (Zhang D. et al., 2018). The study by Kim et al. (2017), which also studied histopathological sequelae of neuroprotection in a model of clip compression, found that lithium reduced locomotor deficits assessed by the Basso, Beattie, and Bresnahan scale, reduced spinal cord leukocyte infiltrates, reduced microglial activation and reduced spinal cord hemorrhage. On the molecular level, lithium was shown to induce the phosphorylation (i.e., inactivation) of glycogen synthase kinase-3β (GSK-3β), a known lithium target, which elevated the GSK-3β target nuclear factor erythroid 2-related factor-2 (Nrf-2) and the Nrf-2 target heme oxygenase-1 (HO-1) (Kim et al., 2017). The Basso, Beattie, and Bresnahan scale is an observation scale that was developed for rats (Basso et al., 1995) and resembles the BMS for mice (Basso et al., 2006).

To exclude examiner influences, we combined the BMS with the horizontal ladder rung test and open field test, showing that lithium indeed induces robust motor coordination improvements associated with brain remodeling that persisted in the post-acute SCI phase for up to 56 dpi. Our study expands the test repertoire for studying motor coordination deficits post-SCI. The development of motor coordination tests in mouse SCI models remains a challenge. The vertical ladder climb test and the grid walking test have been shown to be suitable for studying motor coordination improvements with adequate adjustments, while other tests, such as the inclined plane, the plantar test, and the tail-flick test, were not suitable to discriminate motor coordination changes post-SCI (Pajoohesh-Ganji et al., 2010). The horizontal ladder rung test has originally been established for studying motor coordination recovery following motor cortical lesions (Farr et al., 2006). The open field test is widely used for studying spontaneous locomotor activity in brain injury models (Kilic et al., 2008). It provides useful quantitative readouts, such as the time of mobility, the total distance traveled, mean speed, and rotation movements, which can be analyzed investigator independently in an automated way.

In line with the motor coordination improvements, lithium significantly enhanced evoked responses in the ipsilesional gastrocnemius muscle after electrical stimulation rostral to the spinal cord hemitransection. As such, the latency of EMG potentials was reduced and the EMG potential amplitude was increased by lithium on the lesioned side. EMG recordings provide comprehensive information about motor function after SCI (Tysseling et al., 2013), but motor evoked potentials have so far not been used for studying lithium’s effects on the recovery of the injured spinal cord. The latency of evoked EMG potentials depends on the integrity of myelin sheaths, whereas the amplitude depends on axonal integrity (Schulz et al., 2014). The observation that lithium reduced the evoked potential latency and increased the evoked potential amplitude indicates that axonal regeneration and remyelination were enhanced by lithium. In a rat model of ventral root avulsion and reimplantation of the brachial plexus, lithium has previously been shown to improve the outgrowth and remyelination of peripheral nerves (Fang et al., 2016).

Following spinal cord hemitransection, motor coordination recovery by lithium was closely associated with reduced gray and white matter atrophy rostral and caudal to the hemitransection, increased neuronal survival in the dorsal and ventral horns caudal to the hemitransection, and reduced astrogliosis in the gray matter caudal to the hemitransection. In rat SCI models, improvements of motor coordination impairments closely go in line with the reduced histopathological injury and enhanced white matter integrity (Cao et al., 2005; Dunham et al., 2010). In rat models of a spinal contusion injury, lithium has previously been shown to reduce the size of the lesion cavity (Abdanipour et al., 2019) and increase neuronal survival at the contusion site (Zhang D. et al., 2018). Our present study expands these earlier data by showing that gray and white matter were equally protected by lithium after spinal cord hemitransection. Indeed, significant tissue preservation was noted by planimetry rostral and caudal to the lesion site, which persisted in the long-term. Effects of lithium on perilesional astrogliosis have not been studied after SCI.

By anterograde tract tracing, we finally found that lithium enhanced the regeneration of long-distance axons both proximal and distal to the lesion site. For this purpose, BDA deposits were administered into the lateral spinal cord caudal to the hemitransection, which allowed us to evaluate axonal regeneration of the lateral spinothalamic tract below and above the lesion site. Anterograde tract tracing techniques have previously been used to study plasticity-promoting treatments in mouse and rat models of ischemic stroke (Reitmeir et al., 2011, 2012) and SCI (Guest et al., 1997; Kartje et al., 1999), but had so far not been used for studying lithium effects in the lesioned spinal cord. The combined evidence of this study suggests that lithium induced robust structural spinal cord remodeling resulting in functional tissue recovery. Due to the narrow therapeutic window of lithium, its clinical utility in human stroke patients with cardiovascular risk factors and comorbidities will be low.

## Data availability statement

The original contributions presented in the study are included in the article/Supplementary material, further inquiries can be directed to the corresponding author.

## Ethics statement

The animal study was reviewed and approved by the Istanbul Medipol University Animal Research Ethical Committee.

## Author contributions

ZB, IC, PD, NA, and MB performed the animal experiments and behavioral tests. SB and HK performed EMG analyzes. ZB and MA prepared the histochemical studies. AY and MG performed biochemical analyzes. EK, TD, and DH analyzed data wrote the manuscript. All authors revised and finalized this manuscript.

## Supplementary material

Supplementary Figure 1 Schematic representation of experimental design.

Supplementary Figure 2 Lithium does not influence reactive astrogliosis of the white matter. Reactive astrogliosis in **(A,D)** the dorsal funiculus, **(B,E)** the lateral funiculus, and **(C,F)** the ventral funiculus of the spinal cord assessed by GFAP immunohistochemistry at **(A–C)** the rostrocaudal level above the hemitransection (“Rostral level”) and **(D–F)** the rostrocaudal level below the hemitransection (“Caudal level”) of SCI mice, which were treated with vehicle or lithium (0.2 or 2 mmol/kg/day) for 56 days. Representative GFAP stainings are also shown. Data are mean ± SD values (*n* = 16 animals/group). No significant group differences were detected. Scale bars, 50 μm.
