# Supplementary figures and images for: Lithium promotes long-term neurological recovery after spinal cord injury in mice by enhancing neuronal survival, gray and white matter remodeling, and long-distance axonal regeneration
Source: Front Cell Neurosci. 2022 Nov 11;16:1012523. doi: 10.3389/fncel.2022.1012523 (PMC9693752; doi:10.3389/fncel.2022.1012523)

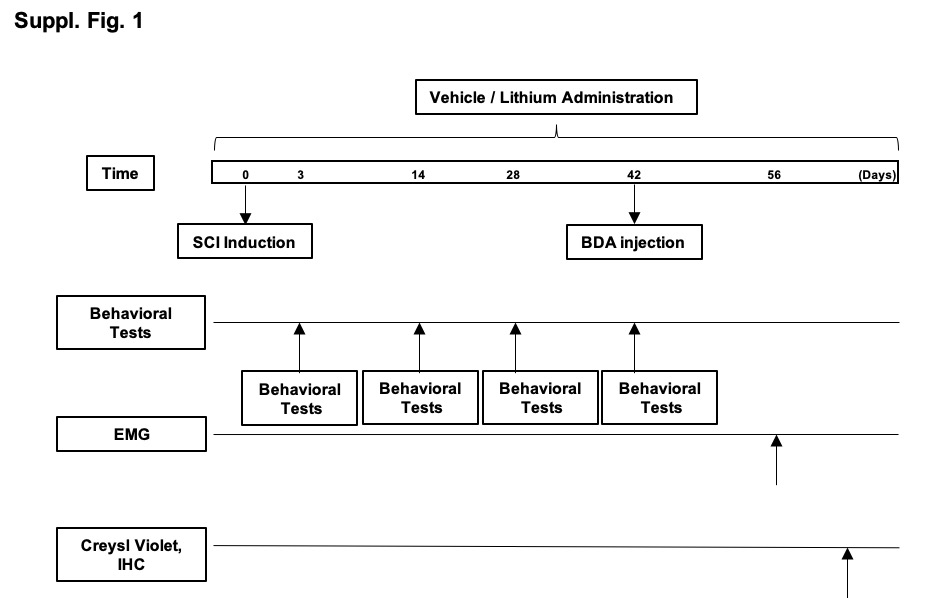

Supplement: Supplementary Figure 1 — Schematic representation of experimental design. [file Image_1.JPEG]

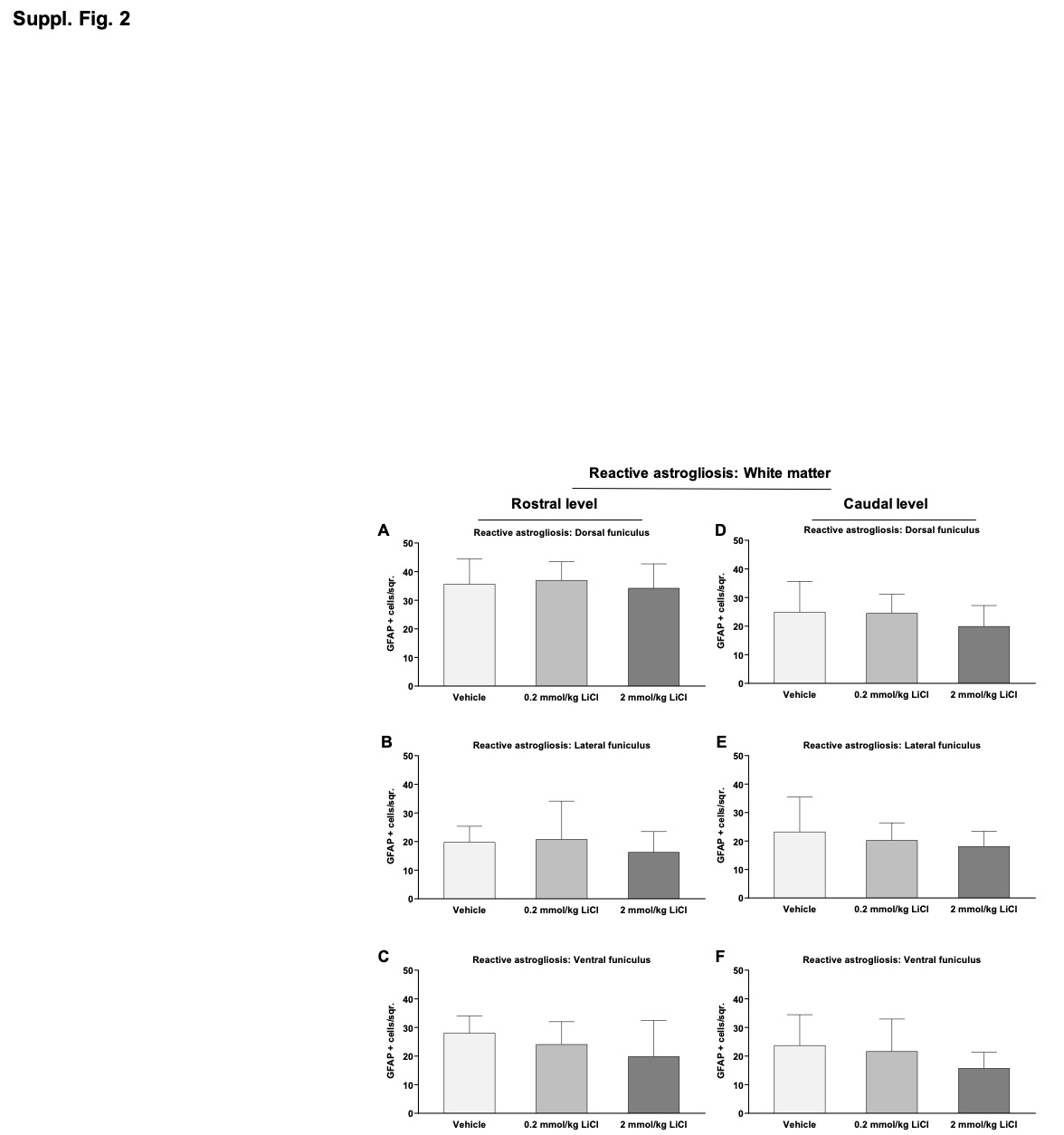

Supplement: Supplementary Figure 2 — Lithium does not influence reactive astrogliosis of the white matter. Reactive astrogliosis in (A,D) the dorsal funiculus, (B,E) the lateral funiculus, and (C,F) the ventral funiculus of the spinal cord assessed by GFAP immunohistochemistry at (A–C) the rostrocaudal level above the hemitransection (“Rostral level”) and (D–F) the rostrocaudal level below the hemitransection (“Caudal level”) of SCI mice, which were treated with vehicle or lithium (0.2 or 2 mmol/kg/day) for 56 days. Representative GFAP stainings are also shown. Data are mean ± SD values (n = 16 animals/group). No significant group differences were detected. Scale bars, 50 μm. [file Image_2.JPEG]
